# Dextran Nanoparticle Synthesis and Properties

**DOI:** 10.1371/journal.pone.0146237

**Published:** 2016-01-11

**Authors:** Iga Wasiak, Aleksandra Kulikowska, Magdalena Janczewska, Magdalena Michalak, Iwona A. Cymerman, Andrzej Nagalski, Peter Kallinger, Wladyslaw W. Szymanski, Tomasz Ciach

**Affiliations:** 1 Warsaw University of Technology, Faculty of Chemical Engineering, Warynskiego 1, 00–645 Warsaw, Poland; 2 NanoVelos Sp. z o.o., Warynskiego 1 / 212, 00–645 Warsaw, Poland; 3 International Institute of Molecular and Cell Biology, Trojdena 4, 02–109 Warsaw, Poland; 4 Faculty of Physics, University of Vienna, Boltzmanngasse 5, A-1090 Vienna, Austria; Brandeis University, UNITED STATES

## Abstract

Dextran is widely exploited in medical products and as a component of drug-delivering nanoparticles (NPs). Here, we tested whether dextran can serve as the main substrate of NPs and form a stable backbone. We tested dextrans with several molecular masses under several synthesis conditions to optimize NP stability. The analysis of the obtained nanoparticles showed that dextran NPs that were synthesized from 70 kDa dextran with a 5% degree of oxidation of the polysaccharide chain and 50% substitution with dodecylamine formed a NP backbone composed of modified dextran subunits, the mean diameter of which in an aqueous environment was around 100 nm. Dextran NPs could be stored in a dry state and reassembled in water. Moreover, we found that different chemical moieties (e.g., drugs such as doxorubicin) can be attached to the dextran NPs via a pH-dependent bond that allows release of the drug with lowering pH. We conclude that dextran NPs are a promising nano drug carrier.

## Introduction

When Pasteur isolated dextran [1] for the first time in 1861, he certainly did not expect that this simple structure, synthesized by bacteria polysaccharide, could find such wide applications in medicine. Although dextran is simply a combination of glucose molecules, it is extensively used in the medical field, primarily as supplementary material that reduces blood viscosity and prevents the formation of blood clots [2]. Moreover, iron-dextran derivatives are used for the treatment of iron deficiency [3], diethylaminoethyl-dextran reduces cholesterol and triglyceride levels, and dextran-sulphate may be used as a coating to increase the biocompatibility of inorganic systems [4]. Dextran has also been applied in nanomedicine, a novel discipline that applies submicron particles for therapeutic and diagnostic purposes. Dextran is used as an alternative for PEGylation to avoid NP and opsonin interactions [4]. Dextran sulfates are used to form NPs via electrostatic interactions with chitosan amine groups [5]. Conjugates of dextran and poly(e-caprolactone) are another popular component of NPs, which are mostly used as micelles that are composed of block polymers that encapsulate the drug inside [6]. Using dextran as an additive to solid lipid NPs also improves the properties of drugs that are delivered orally (e.g., ibuprofen) [7]. Dextran was also used to protect and stabilize unique structure of protein (such as albumin, streptokinase, asparagines, insulin, hemoglobin) [8–10]. Dextran supplementation allows to extend protein biodistribution time, reduces protein’s immunogenicity while keeping they high activity.

Considering dextran’s widespread use and effective metabolism and clearance in the liver and spleen [11], it is tempting to exploit dextran as not only a supplementary material but as a nanosystem backbone. Thus, we investigated whether dextran can form a stable NP backbone, particularly how various modifications of dextrans with several molecular masses influence NP formation. We also tested several chemical modifications to optimize the stability of NPs.

## Materials and Methods

### 2.1 Nanoparticle synthesis

#### 2.1.1 Synthesis of polyaldehydodextran

The dextrans (Nobilus, Kutno, Poland; isolated from *Leuconostoc mesenteroides*, M_W_ = 15, 40, 70, and 500 kDa) were oxidized according to a modified protocol of Muangsiri and Fuentes [12,13]. Briefly, 10 g of each dextran was dissolved in 200 ml distilled water. Subsequently, sodium metaperiodate (Sigma-Aldrich) was added in molar ratios of 1:20, 1:10, 1:7.5, and 1:5 (IO^-^_4_/glucose units). For details, see Table 1. The solutions were stirred in the dark at room temperature for 1 h. The reactions were quenched by the addition of ethylene glycol (Chempur, Piekary Slaskie, Poland). The solutions were dialyzed against distilled water using a dialysis membrane bag (Carl-Roth, MWCO 12–14 kDa) for 3 days. The final products were dried in an incubator at 50°C for 24 h. The number of aldehyde groups in polyaldehydodextran (PAD) were determined using a modified hydroxylamine hydrochloride method [14] with unmodified dextran as a reference.

**Table 1:** Amount of substances used in dextran oxidation.

Molar ratio	1:20	1:10	1:7 5	1:5
Dextran [g]	10	10	10	10
NaIO_4_ [g]	1.2	2.3	3.5	4.9
Ethylene glycol [ml]	0.6	1.2	1.9	2.45

#### 2.1.2 Synthesis of dextran nanoparticles (Dex-NPs) and with doxorubicin (Dox-NPs)

**Fig 1:**
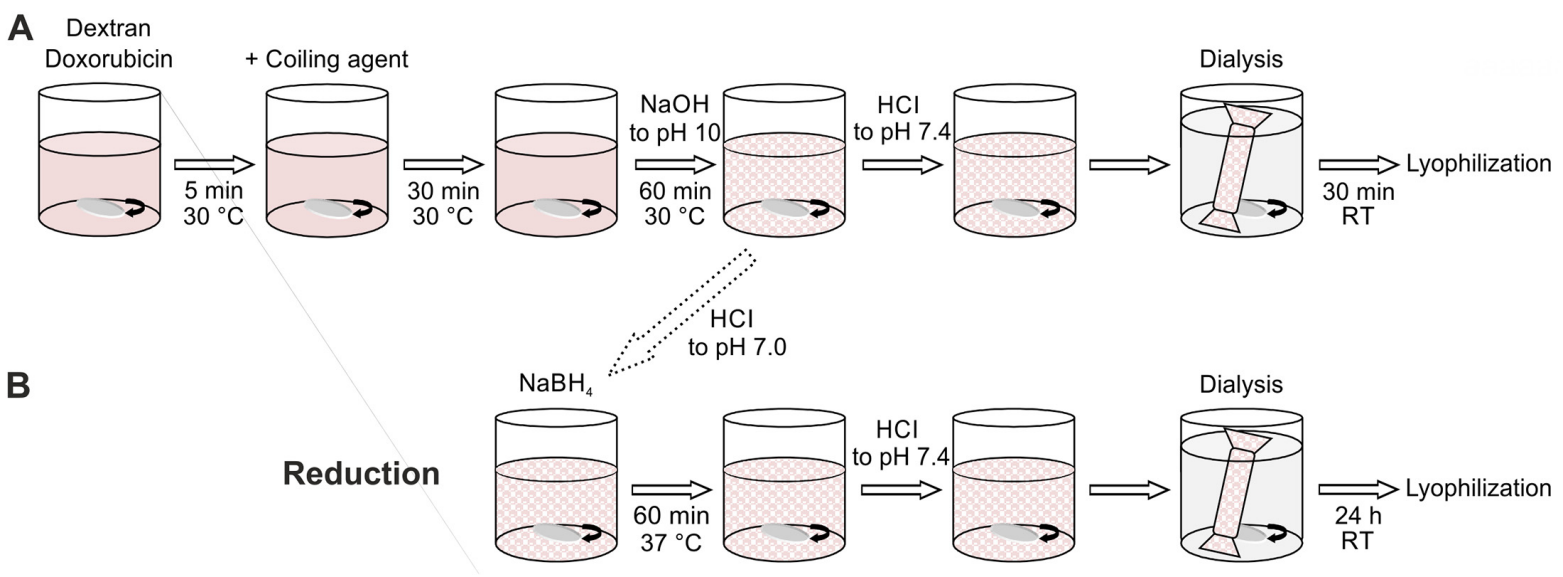
Diagram of nanoparticles synthesis strategy.

One gram of dried PAD was dissolved in 10 ml of distilled water at 30°C, and prewarmed coiling agent solution (Table 2) was added. For Dox-NPs synthesis, 0.01 g/ml of doxorubicin aqueous solution was added 5 min before the coiling agent. The mixture was constantly stirred at 30°C for 30 min. The pH was measured and increased with 0.5 M sodium hydroxide within 60 min until the solution reached pH 10. Finally, the pH of the mixture was decreased to 7.4 with HCl, and the obtained Dex-NPs solution was dialyzed against distilled water for 30 min. Dex-NPs were lyophilized using dextran with the corresponding molecular weight as a cryoprotectant. Nanoparticles with different dextran molecular weights and coiling agent chain lengths (hexylamine, octylamine, dodecylamine, and benzylamine) were synthesized the same way. The schematic representation of the NPs synthesis is presented on Fig 1A.

**Table 2:** Amount of substances in NP synthesis for 1 g dextran.

**Dextran MW [kDa]**	15		40					70												500	
**Oxidation degree [%]**	5		5					5						10		15		20		5	
**Substitution degree [%]**	50	100	11	22	33	55	100	11	22	33	50	55	100	50	100	50	100	50	100	50	100
**Coiling agent [mmol]**	0.41	0.90	0.09	0.18	0.27	0.45	0.90	0.09	0.18	0.27	0.41	0.45	0.90	0.90	1.80	1.35	2.70	1.80	3.60	0.45	0.90
**Doxorubicin [mmol]**	-	-	-	-	-	-	-	-	-	0.08		0.08								-	-
**Alanine [mmol]**	0.12	-	0.63	0.54	0.44	0.1	-	0.63	0.54	0.44	0.12	0.1	-	0.12	-	0.12	-	0.12	-	0.12	-
**Cryoprotectant [kDa]**	15		40					70												500	

#### 2.1.3 Synthesis of dextran nanoparticles with reduced bonds (rDox-NPs)

Bonds between dextran aldehyde groups and amine groups of doxorubicin were reduced at 37°C for 1 h with NaBH_4_ (Sigma-Aldrich) EtOH solution. The pH of the mixture was set to 7.4 with HCl, and the obtained rDox-NP solution was dialyzed against distilled water for 24 h (Fig 1B). The reduction of the amine bond results in the rDox-NPs formation. As the release of the doxorubicin from rDox-NPs is not possible, rDox-NPs can be used for the calculation of the drug encapsulation efficiency. Concentration of doxorubicin that remained unbound after the synthesis was measured after 24h dialysis in PBS (pH 7.4) solution by spectrophotometric analysis (Epoch^TM^, BioTek^®^ Instruments, Winooski, Vermont, USA) at 490 nm.

### 2.2 Dex-NP diameter and size distribution

The mean diameter and size distribution of the Dex-NPs were determined with an LM 10 –HS Nanosight instrument (Malvern Instruments Ltd.) with 405 nm laser scattering and Nanoparticles Tracking Analysis software. The measurements were replicated at least three times.

### 2.3 Lyophilization

Prior to drying, the Dex-NPs were frozen at -40°C and then lyophilized for approximately 25 h. As a cryoprotectant, dextran was added at a 1% (w/w) ratio (Table 2).

### 2.4 *In vitro* drug release

Doxorubicin release from the NPs was evaluated using the dialysis tube diffusion technique. Dry drug-loaded NPs (lyophilized) were suspended in water for self-assembly (final drug concentration 1 mg/ml) under gentle stirring for 45 min. As a control of doxorubicin release rate from dialysis bag water solution of doxorubicin was used (doxorubicin concentration 1 mg/ml) Ten milliliters of the suspension was then placed inside the dialysis bag (Carl Roth, MWCO 12–14 kDa) in glass flasks that contained 100 ml phosphate-buffered saline (PBS), pH 7.4 and 5.5, as release media. The samples were kept at 37°C and light-protected. At selected time points between 0.5 and 384 h, 1.6 ml of the release media was withdrawn and replaced with fresh PBS, and the concentration of doxorubicin that was released from the NPs was analyzed using an ultraviolet (UV)-visible microplate spectrophotometer (Epoch^TM^, BioTek^®^ Instruments, Winooski, Vermont, USA) at 490 nm. The standard calibration curve of absorbance as a function of doxorubicin concentration was studied at 490 nm with the UV-visible microplate spectrophotometer.

### 2.5 Nanoparticle composition analysis

For the assessment of the possible internal composition of NPs (i.e., the number of modified dextran chains that participated in the formation of a single NP) aerosol technology methods were employed [15–17]. Dex-NPs were dispersed in an electrically conductive liquid and transferred to the aerosol phase by electrostatic atomization [18,19] by means of a nanoelectrospray generator (TSI Inc., Mod. 3480, Shoreview, MN, USA). The so produced monosized droplets from the solution containing the desired material had a diameter of approximately 150 nm. The electrospray conditions were adjusted in such a way that single droplet contained at most a single analyte molecule. The droplets were then dried and brought into known charge equilibrium using a bipolar diffusion charging process to obtain NPs which were either electrically neutral or singly charged. Consequently, charged NPs were size-separated in a custom-built (Tapcon & Analysesysteme, Salzburg, Austria) differential mobility analyzer (DMA) according to their electrical mobility [17], and the selected fraction was counted using a Condensation Particle Counter (CPC). From this data the mobility-equivalent diameter was calculated and used to determine the number of dextran chains per NP. Serial dilutions of NPs, beginning from 10 mg/ml in 20 mM aqueous (water for analysis; Merck) ammonium acetate (Sigma-Aldrich) solution, were measured. If the droplet did not contain analyte no signal was recorded as ammonium acetate sublimates during droplet drying process.

### 2.6 Scanning electron microscopy (SEM) analysis of Dex-NPs

Visualization of Dex-NPs was performed using novel electron imaging method applying ionic liquids: Dex-NPs were dissolved in ultra pure water to prepare stock solution to the concentration of 1g/ml. NPs stock solution was incubated with 5% solution of HILEM^®^ IL 1000 in proportion 1:1 for 2–3 min. After incubation samples were put on the TEM grid. Images were collected on scanning electron microscope (SEM) SU8320 (Hitachi, Japan) instrument in scattered electrons (SE) mode and dark field STEM (DF) mode with accelerating voltage of 30 kV.

### 2.7 Cell culture

HeLa cells (ATCC) were seeded on cover glasses in 24-well plates and cultured in Dulbecco’s Modified Eagle Medium (DMEM) supplemented with 2 mM L-glutamine, 1% penicillin-streptomycin, and 5% fetal bovine serum (FBS; all reagents from Gibco). After incubation for 24 h at 37°C in 5% CO_2_, the culture medium was replaced with fresh medium supplemented with doxorubicin, Dox-NPs, or rDox-NPs normalized to the equal final concentration of doxorubicin of 10 μg/ml. The cells were incubated with NPs for 30 min. Subsequently, the media were removed and replaced with fresh media. The cells were incubated for 2 h and fixed with 4% paraformaldehyde for 10 min. After fixation, the cells were rinsed three times with PBS and mounted with ProLong Gold Antifade Mountant with DAPI (Life Technologies) according to the manufacturer’s protocol. Photomicrographs were recorded using a Zeiss 710 NLO confocal system equipped with a 40c/1.3 oil-immersion objective. Five Z-stacks (0.5 μm) were collected and used for Maximum Intensity Projections.

Cytotoxicity tests were made according to the procedure from International Standard Operation (ISO) 10993–5 “Tests for *in vitro* cytotoxicity”. The cytotoxicity of the nanoparticles was tested by MTT viability assay against HeLa cells (ECACC). HeLa cells were seeded in 96-well plates (1 x 10^4^ cells/well) in 100 ml of Dulbecco’s Modified Eagle medium (DMEM) supplemented with 10% fetal bovine serum (FBS) for 24 h at 37C in a humidified 5% CO2-containing atmosphere. After 24 h the media in 96 well plate was aspirated and replaced by 100 μl media containing different concentrations of doxorubicin or doxorubicin-loaded NPs. The cells were incubated for additional 24 h. The medium was aspirated and replaced by 50 μl 3-(4,5- dimethylthiazol-2-yl)-2,5-diphenyltetrazoliumbromide (MTT) solution (1mg/ml from Sigma Aldrich). The cells were incubated for 2 h before the medium was aspirated. The MTT-formazan generated by live cells was dissolved in 100 μl isopropanol and the absorbance of each well was measured using a microplate reader at a wavelength of 570 nm (reference 650 nm). The relative cell viability (%) was determined by comparing the absorbance in each well at 570 nm with blanks. Data are presented as average ±SD. Five concentrations of Dex-NPs (55% substitution) Dox-NPs and rDox-NPs were examined, which was equivalent to 1, 10, 100, 1000, 10000 nM doxorubicin.

## Results

### 3.1 Dex-NP synthesis and dextran oxidation

**Fig 2:**
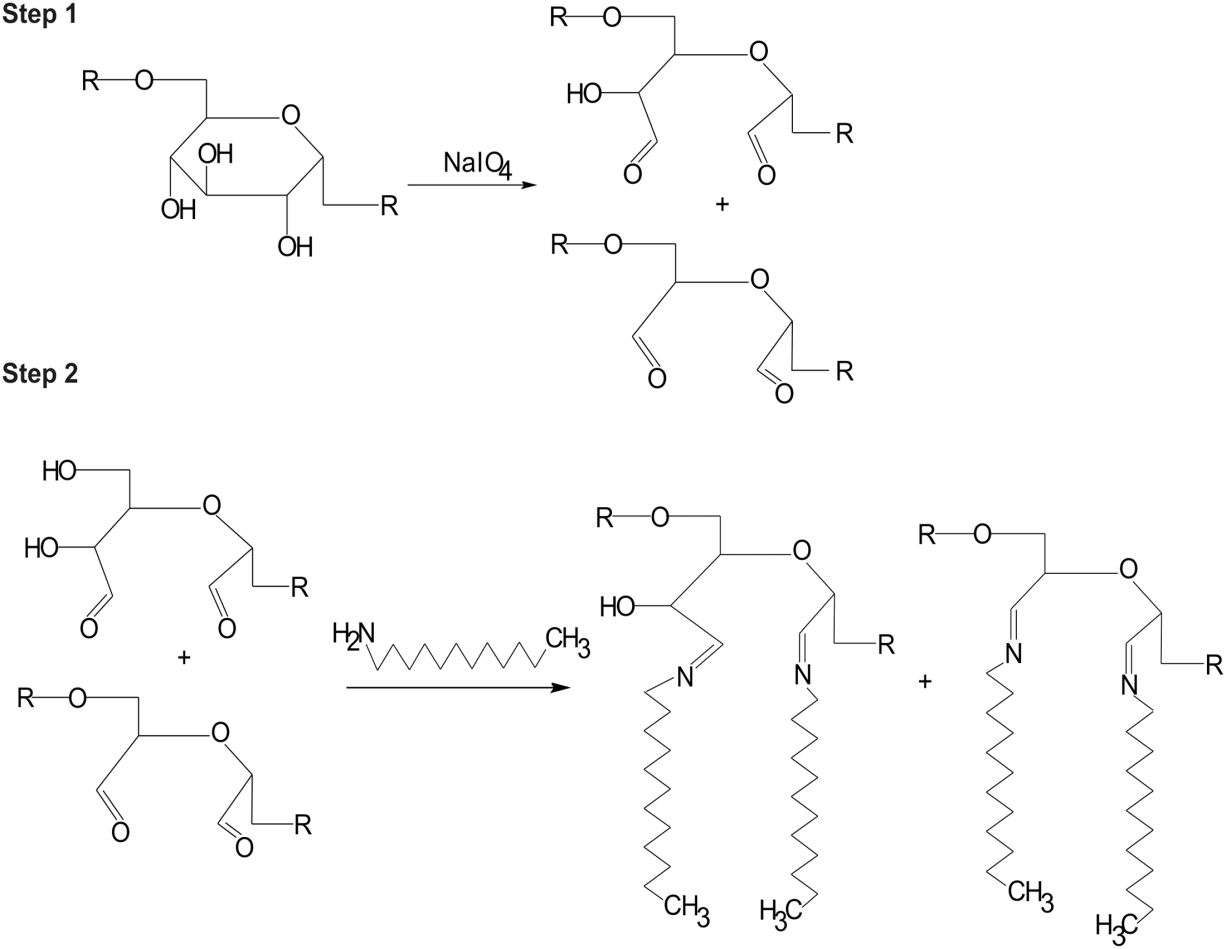
Scheme of dextran modifications. Step 1: Oxidation, leading to opening of the glucose rings. Step 2: Schiff base formation between glucose carbonyl group and aliphatic amine.

To obtain Dex-NPs, single dextran molecules must undergo numerous modifications. To create covalent bonding with amines, dextran is oxidized to aldehyde groups while keeping the polysaccharide chain intact (Fig 2 (Step 1)). The amount of NaIO_4_ that was added to the reaction was calculated to oxidize 5% of the dextran glucose rings. Dextran oxidation is controlled and confirmed by determination the number of aldehyde groups in dextran backbone in comparison non oxidized dextran. The number of aldehyde groups in the resulting product reached 7% ± 0.3% (Table 3). The discrepancy between the expected and obtained oxidation degrees was consistent with earlier reports [12], in which the authors explained that every molecule of periodate causes the formation of approximately 1.7 aldehyde groups. Under our experimental conditions, however, the oxidation factor deviated between 1.2 and 1.5, depending on the reaction conditions (Table 3). Thus, we contend that the degree of oxidation must be monitored every time for every theoretically predicted dextran oxidation reaction.

**Table 3:** Comparison of theoretically predicted and experimentally measured dextran oxidation degrees.

Theoretical oxidation degree [%]	Measured oxidation degree [%]	Oxidation factor [–]
5	7	1.5
10	13	1.3
15	17.5	1.2
20	23	1.2

Schiff reaction was already applied in vascular prosthesis sealing and products proved its biocompatibility and hydrolytic degradation [20] and we decided to exploit the ability of Schiff base formation between the active carbonyl groups present in PAD and amino groups of the coiling agent (aliphatic amine) or drug, resulting in pH-sensitive carbon-nitrogen double bonding (Fig 2 (Step 2)) during nanoparticle synthesis. Moreover, introducing of hydrophobic moieties to dextran chains enable NP formation in the self-assembly process. The formation of connection between coiling agent and dextran is monitored during reaction visually. When coiling agent does not form Schiff base it precipitates from water solution at pH = 9, which can be observed by the turbidity of the solution. Such observation is not observed when the Schiff base is formed.

### 3.2 Influence of synthesis parameters on NP diameter

**Fig 3:**
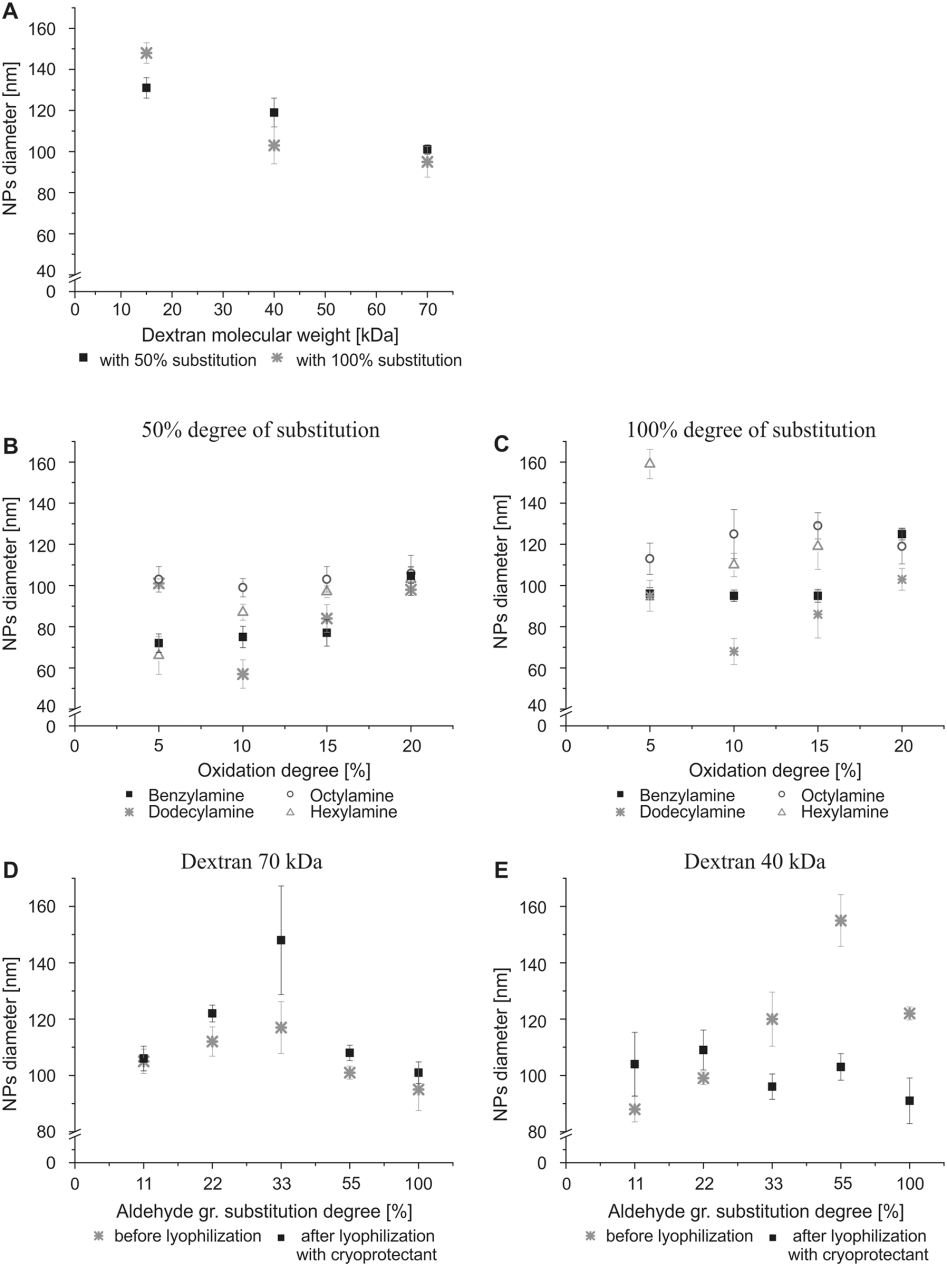
Influence of synthesis parameter modifications on Dex-NP diameter. (A) Diameter of Dex-NPs (non lyophilized) synthesized from dextran with 15, 40, and 70 kDa molecular weights and 50% and 100% degrees of substitution, dodecylamine as a coiling agent, 5% oxidation degree. Dextran with 70 kDa and (B) 50% degree of substitution and (C) 100% degree of substitution with various coiling agents. Percentage of degree of substitution of dodecylamine on (D) 70 kDa dextran; 5% oxidation degree and (E) 40 kDa dextran; 5% oxidation degree. Mean *n* = 3 ± SD.

The key NP features that determine their potential use in nanomedicine are their diameter and size distribution. Nanoparticle synthesis conditions strongly influence these features; thus, we performed a series of experiments to modify key parameters, such as dextran molecular weight, dextran oxidation degree, coiling agent, and degree of aldehyde group substitution (Fig 3).

### 3.3 Influence of dextran molecular weight on NP diameter

Four Dex-NPs with four PAD molecular weights (15, 40, 70, and 500 kDa) and a 5% degree of oxidation were synthesized. The amount of dodecylamine that was added was calculated to substitute 50% or 100% of the introduced aldehyde groups on the dextran backbone (see Table 2). For 15, 40, and 70 kDa PAD, the size of the NPs decreased as the molecular weight of dextran increased (Fig 3A). The synthesis of NPs from 500 kDa dextran did not work under the present experimental conditions, likely because of the high viscosity of the dextran solution (we tried a 1% dextran concentration, which resulted in a significant deviations of the measured NP diameter, disqualifying them from possible applications and further analysis: the obtained NPs had a diameter of 180 ± 35 nm for a 50% degree of substitution and diameter of 143 ± 42 nm for a 100% degree of substitution).

For further analysis we chose PADs of 40 and 70 kDa due to the converge size distribution for both substitution degrees, indicating the sustained NPs characteristic in case of full substitution of aldehyde groups.

### 3.4 Influence of coiling agent type and oxidation degree on NP diameter

Dex-NPs were synthesized from 70 kDa PAD with four increasing degrees of oxidation ranging from 5% up to 20% with four coiling agents (Fig 3B and 3C). The amounts of the coiling agents that were added were appropriate for substitution of 50% or 100% of the aldehyde groups in the dextran backbone (see Table 2). Nanoparticle diameters were measured after synthesis, followed by 30 min dialysis. As we finally aim to exploit NPs as a drug carriers we will eventually substitute all the remaining aldehyde groups with drugs and reach 100% degree of substitution. Thus we looked for the conditions where the full saturation of aldehyde groups does not impair NPs structure. For 50% and 100% degrees of substitution, the trend of the NPs diameter change was retained by benzylamine and dodecylamine whereas in the case of octylamine and hexylamine the trends were not concurrent. For further analysis, dodecylamine was chosen because of its lowest *in vivo* toxicity (i.e., the LD_50_ in rats is 1020 mg/kg) compared with other coiling agents (the LD_50_ for benzylamine in rats is 552 mg/kg; the LD_50_ for hexylamine in rats is 240 mg/kg; the LD_50_ for octylamine in rats is 250 mg/kg).

### 3.5 Influence of percentage degree of substitution on NP diameter

Dex-NPs from 70 and 40 kDa PAD with a 5% degree of oxidation and 11%, 22%, 33%, 55%, and 100% degrees of substitution were synthesized. The sizes of the NPs were measured at two time points: after synthesis followed by 30 min of dialysis and after lyophilization followed by the rehydration process.

For 70 kDa PAD with an increasing amount of coiling agent added (% substitution with dodecylamine), the NP size initially increased (until a 33% degree of substitution) and then decreased (Fig 3D). The average sizes of the Dex-NPs for the 11% degree of substitution were around 100–110 nm while size distribution measurements resulted with either a single or bimodal peak. We assumed that the concentration of dodecylamine at the 11% degree of substitution caused unstable Dex-NP formation. Instability can arise from the combination of physical and chemical properties. Amine chains are far apart and do not interact with each other strongly enough to form a stable structure or the reaction products undergo further conversion to the next step of the Maillard reaction (i.e., the dissolution of Amadori products when short hydrolysis products, such as methylglyoxal and diacetyl, are formed). The measurements of Dex-NPs for the 22% degree of substitution resulted in single peaks, indicating a NP diameter of 112 ± 5.2 nm, for 33% of 117 ± 9.2 nm, for 55% of 101 ± 2.3 nm and for 100% of 95 ± 7.5 nm before lyophilization and of 122 ± 3 nm, 148 ± 19.3 nm, 108 ± 2.7 nm and 101 ± 3.8 nm after lyophilization, respectively. Large error bars for Dex-NPs of 33% substitution before lyophilization are caused by high differences between particular NPs batches synthesis. We assume that in this particular formulation the synthesis conditions are less stable.

For 40 kDa PAD, the NP diameters before lyophilization increased with the degree of substitution from 88 up to 155 nm, with the exception of the 100% degree of substitution, where the diameter of NPs was 122 ± 2.3 nm (Fig 3E). After lyophilization, this trend disappeared, and the size of the NPs ranged from 90 to 120 nm.

We assume that the increase of NPs diameter before and after lyophilization (Fig 3D and 3E) can result from different water incorporation into the NP structure during rehydration as water removal may influence packaging of dextran chains.

### 3.6 Doxorubicin encapsulation efficiency

Drug encapsulation efficiency was calculated as the ratio of doxorubicin that was chemically bound to the NPs to the total amount of the doxorubicin used in the synthesis. To distinguish between Doxorubicin fraction that was not bound to the NPs during synthesis and Doxorubicin fraction that is released from NPs upon release rDox-NPs were used. Doxorubicin that was not firmly connected to nanoparticles during synthesis migrated during 24 h dialysis (after synthesis) out of the dialysis bag to the dialysat. The concentration of doxorubicin in dialysat was used for the spectroscopic measurement of the unbound amount of doxorubicin. Finally, the encapsulation efficiency ranged between 92–96% (3 independent synthesis).

### 3.7 Lyophilization

**Fig 4:**
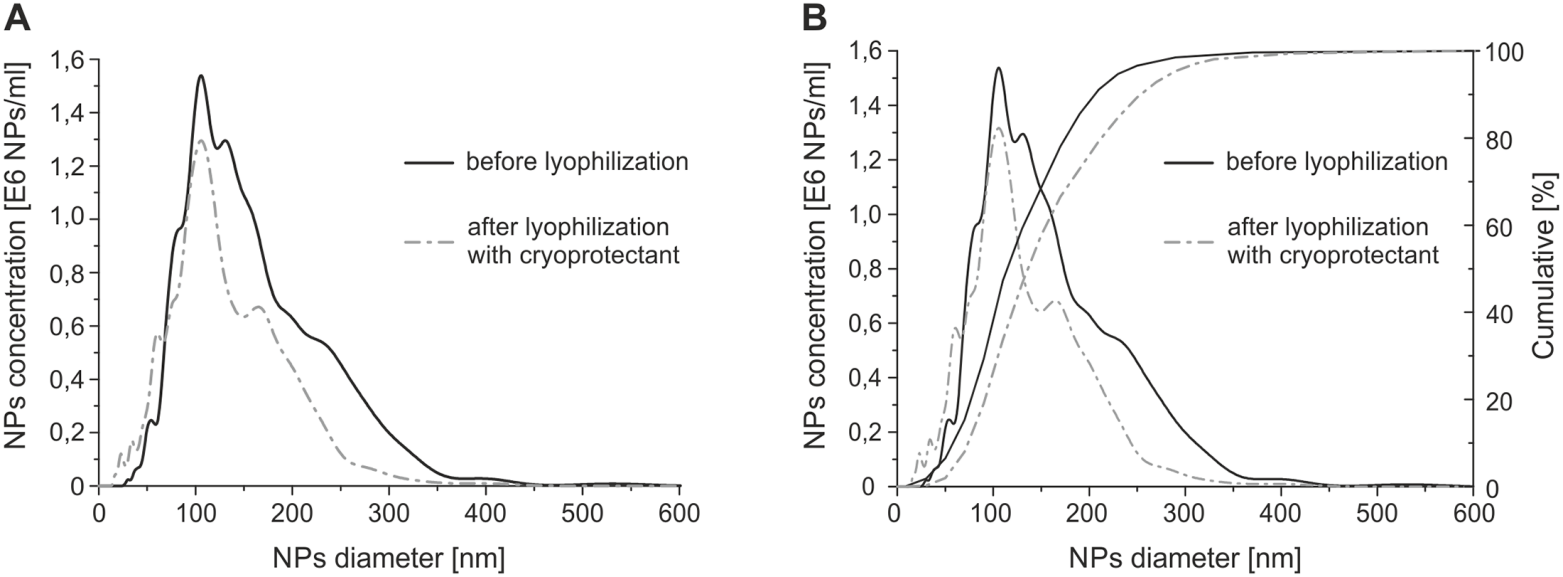
A) Size distribution curve and B) cumulative size distribution of Dex-NPs before and after lyophilization and rehydration. Graph represents size distribution of Dex-NPs for 55% substitution degree.

We used lyophilization for the long-term storage of Dex-NPs. Due to the fact, that almost every drug should be transported and stored after manufacturing and before administration, we assume that nanoparticles will undergo the lyophilization and rehydratation steps. The use of a cryoprotectant is recommended to overcome uncontrolled aggregation and destruction of the NP structure [21]. Many cryoprotectants are commonly used for NP lyophilization, such as glucose, sucrose, lactose, mannitol, poly(vinyl alcohol), and dextran. Few features of the lyophilized product need to be verified: (*i*) rapid rehydration, (*ii*) unaffected NP size distribution, (*iii*) long stability of the desiccated product, and (*iv*) humidity < 2% [21]. In our case, dextran with the same molecular weight as during synthesis served as the cryoprotectant. The NP diameter distribution remains concurrent after the lyophilizatation and rehydratation process (Fig 4) Diameter distribution measurement revealed the population of NPs that diameter was greater than 200 nm as not greater than 20%. This “tail” reflects the natural chain length distribution of the raw dextran used for synthesis.

### 3.8 *In vitro* drug release

**Fig 5:**
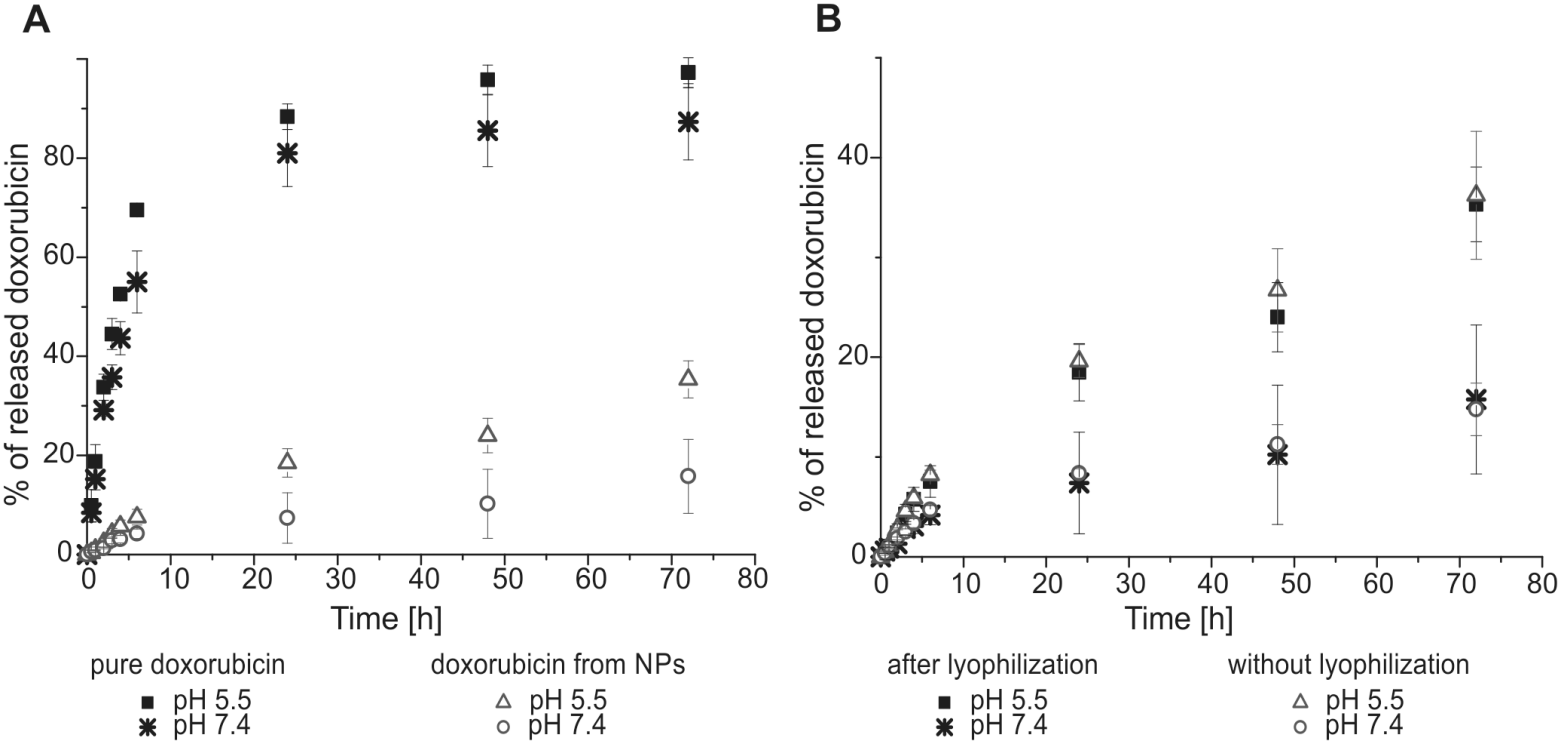
Doxorubicin release rate in PBS. (A) Comparison of doxorubicin release from the dialysis bag in the control group (raw doxorubicin) and Dox-NPs (rehydrateded) in the PBS solution at two pHs (5.5 and 7.4) at 37°C. (B) Effect of NP lyophilization on the rate of doxorubicin release in PBS solution at pH 5.5 and 7.4 at 37°C. Mean *n* = 3 ± SD.

The *in vitro* drug release from doxorubicin loaded nanoparticles was monitored as a function of released doxorubicin in PBS buffer at pHs 7.4 and 5.5 at 37°C over time. We also tested whether a difference exists in the release of doxorubicin from NPs with and without lyophilization. After 24 h, the amount of doxorubicin that was released from NPs was approximately 18.5% at pH 5.5 and 7.4% at pH 7.4, whereas the amount of doxorubicin that was released in the control group (raw doxorubicin) was 88.4% and 81.0%, respectively (Fig 5A). At the initial time points we did not observed an undesirable burst effect in *in vitro* drug release. Over 72 h (Fig 5B) and in later time points (S1 Fig) of drug release monitoring, we noticed no significant effect of NPs lyophilization on the rate of doxorubicin release indicating sustained release.

### 3.9 Nanoparticle composition

**Fig 6:**
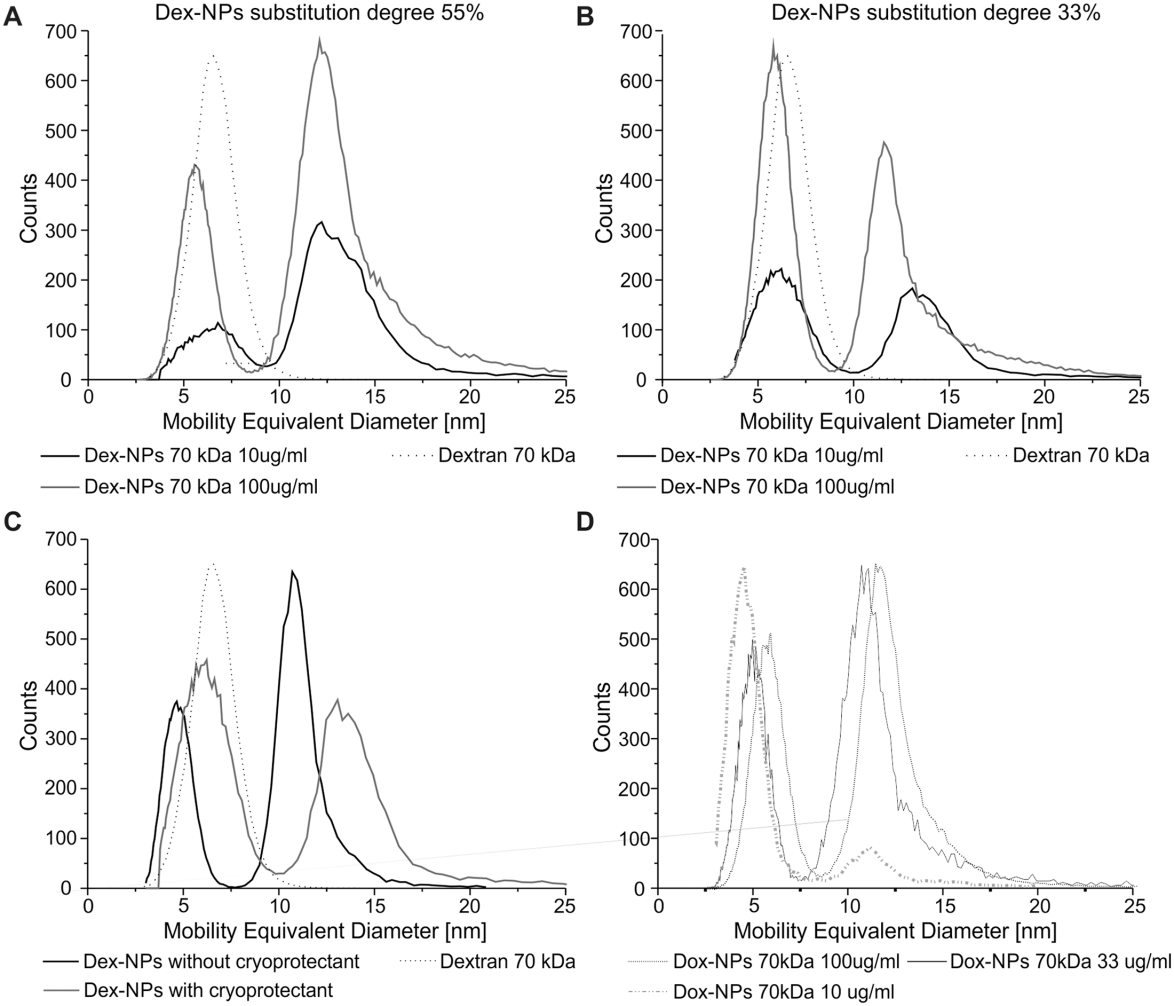
Mobility-equivalent diameter distribution of nanoparticles. Dex-NPs with a 55% (A) and 33% (B) degree of substitution (10 μg/ml and 100 μg/ml) referred to pure dextran 70 kDa (7μg/ml). (C) Dex-NPs with a 33% degree of substitution (10μg/ml) with and without cryoprotectant referred to pure dextran 70 kDa (7μg/ml). (D) Dox-NPs with a 33% degree of substitution (10 μg/ml; 33 μg/ml and 100 μg/ml). Mean n = 5.

Dex-NPs with 70 kDa dextran and two degrees of substitution (55% and 33%) as well drug-containing NPs (Dox-NPs with 70 kDa dextran) with a 33% degree of substitution were examined to determine the NP composition using aerosol techniques (Fig 6).

The Dex-NP suspensions show a bimodal distribution (Fig 6) when compared to the pure 70 kDa dextran that showed a mean mobility-equivalent diameter of 6.6 nm. Peaks at mobility-equivalent between 5.6 and 6.8 nm originated mostly from pure dextran, which was added to the samples as a cryoprotectant (5% w/w), and single subunits of modified dextran. Shift between modified and original dextran mobility equivalent diameters came from different packing/density or different shape of aerosol nanoparticles. Peaks of around 12 nm represent Dex-NPs.

The presence of solely those two peaks suggests a tendency to form NPs structure while minimizing the free energy. Mobility of aerosol particles is proportional to its geometric diameter. Volume of particles is proportional to the third power of diameter. The diameter ratio of aerosol particles formed from Dex-NPs to the aerosol particles formed from single chains of dextran is slightly above 2 (12/5,5). We can calculate that volume of Dex-NPs is approximately 10 times higher than the volume of particle composed of single dextran chains. Thus we postulate that nanoparticles are composed of approximately 10 modified dextran subunits. At higher concentrated NPs solution equilibrium between NPs and free dextran subunits shifted towards the NPs formation, what reveals micelle like properties of obtained NPs (Fig 6A). The binding constant of modified dextran in NPs depends on the degree of substitution of dextran chains: the more hydrophobic groups (Fig 6A), the more dextran organized in NPs and the less free modified dextran chains present in the solution (when compared to Fig 6B). The attachment of doxorubicin does not influence the bimodal character of the peaks distribution (Fig 6D) but the lowest measured concentration of 10 μg/ml promotes Dox-NP dissociation into single dextran chains. The presence of cryoprotectant (Fig 6C) does not influence the bimodal distribution but has an impact on mobility equivalent diameter—the phenomena which needs further investigation.

### 3.10 SEM visualization of Dex-NPs

**Fig 7:**
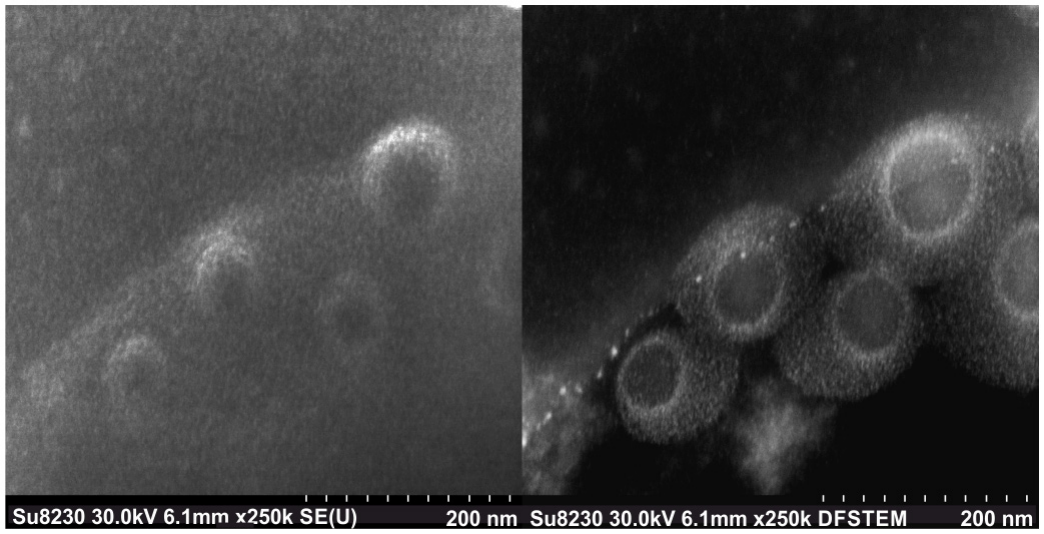
SEM visualization of Dex-NPs nanoparticles in two detection modes SE and DF STEM.

Images of Dex-NPs revealed formation of spherical nanoparticles (Fig 7). Light shadows around Dex-NPs observed in STEM dark field detection mode are caused by water repulsed from Dex-NPs interior by ionic liquids. This phenomenon was not observed in SE detection mode.

### 3.11 *In vitro* relevance

**Fig 8:**
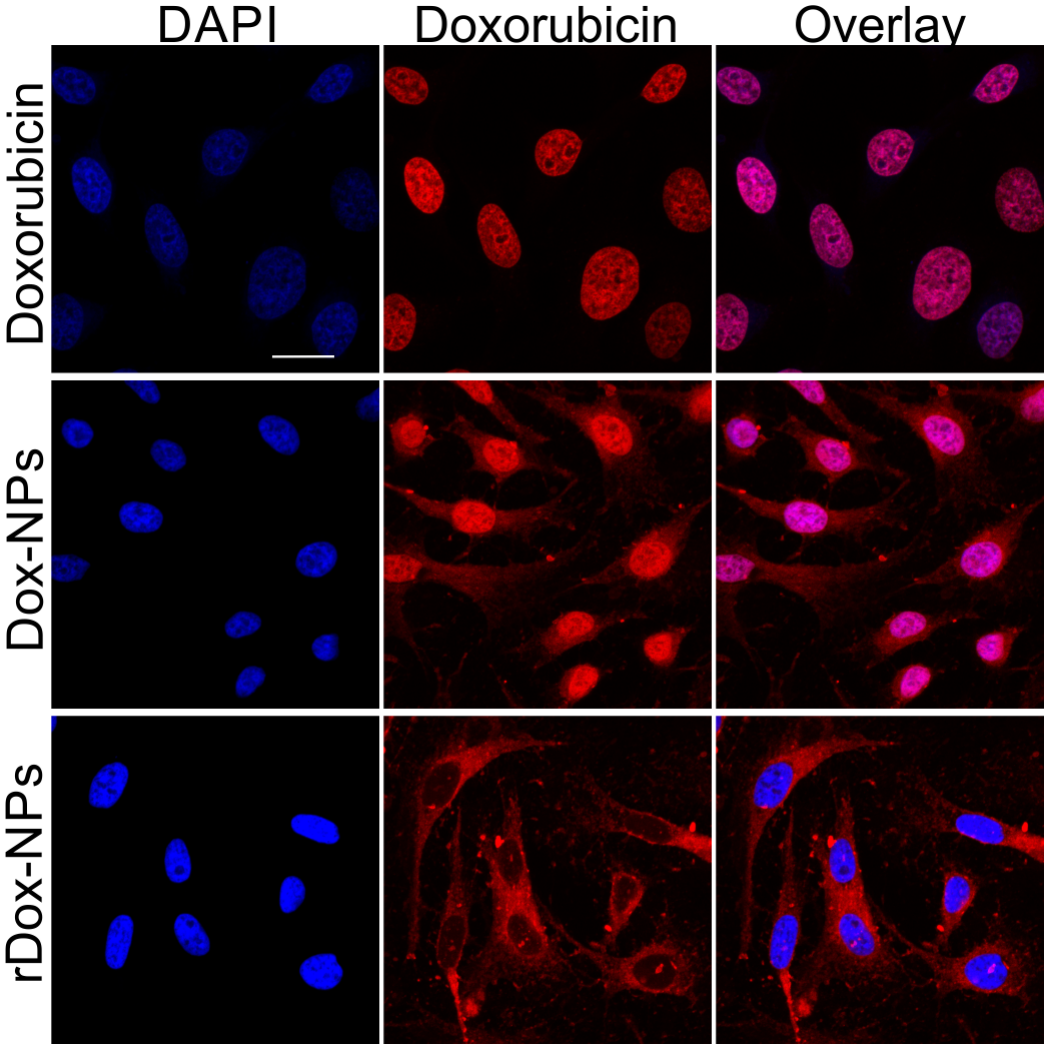
Dox-NPs release doxorubicin within cells. Photomicrographs of fixed HeLa cells incubated with free doxorubicin, Dox-NPs, and rDox-NPs. Cell nuclei were stained with DAPI (blue). Red color arises from natural fluorescence properties of doxorubicin. Scale bar = 20 μm.

To verify whether doxorubicin can be released from NPs intracellularly, we monitored the fluorescence of doxorubicin within HeLa cells that were incubated with free doxorubicin (control), Dox-NPs, and rDox-NPs (Fig 8).

Free doxorubicin intercalates into the DNA of cell nuclei. The fluorescence signal of doxorubicin delivered to the cells via Dox-NPs was observed in the cell nuclei and in the cytoplasm. The cytoplasmic signal arose from doxorubicin bound to the NPs (unreleased fraction). To verify whether the nuclear signal arose from doxorubicin that was already released from the NPs (i.e., Dox-NPs did not enter cell nuclei), we chemically reduced the bond between doxorubicin and NPs to make doxorubicin release impossible. In fact, the signal that arose from rDox-NPs was undetectable within cell nuclei (only in cell cytoplasm), confirming that the nuclear signal in the case of Dox-NPs arose from released doxorubicin. This experiment also confirmed that whole NPs entered into the cell cytoplasm. Moreover, no drug release after chemical reduction of the bond between doxorubicin and nanoparticles confirmed Schiff base formation.

**Fig 9:**
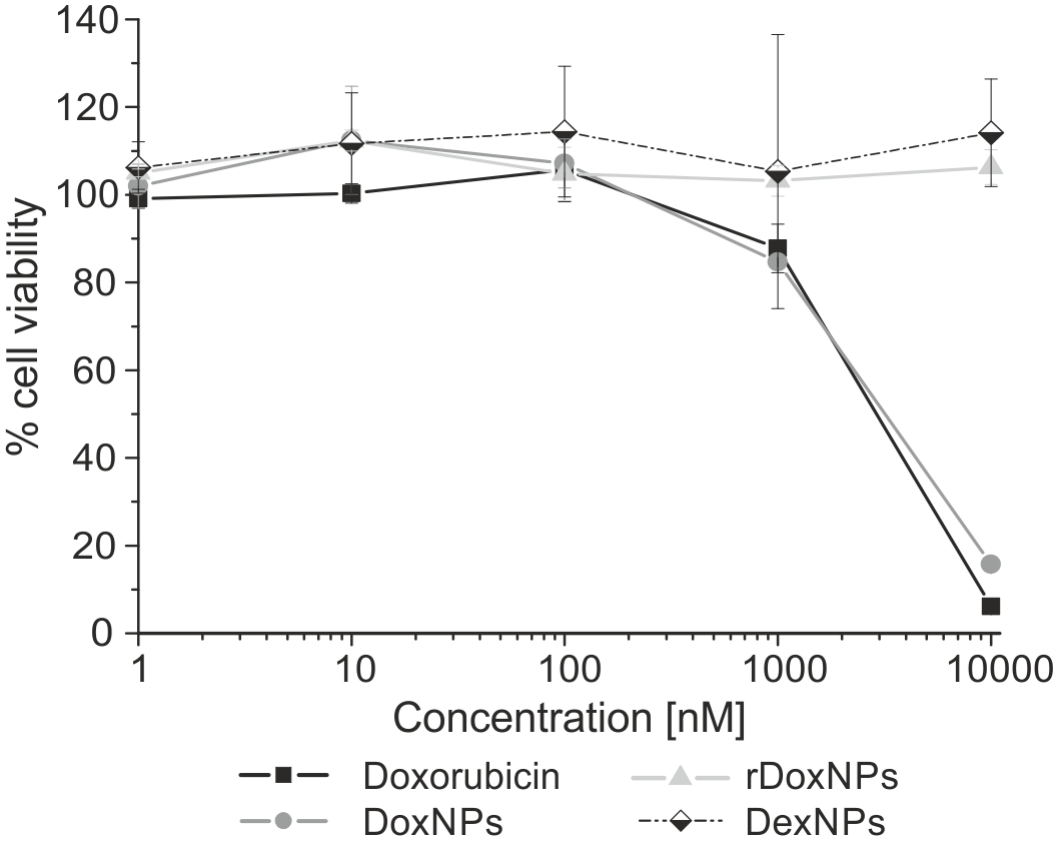
HeLa cell viability after 24h incubation with doxorubicin, Dex-NPs, Dox-NPs and rDox-NPs.

No toxicity of Dex-NPs and rDox-NPs was observed for the concentration in range 1–10000 nM (equivalent to doxorubicin) in MTT assay (Fig 9). Dox-NPs reduced cell viability at the concentration of 1000 nM by 15% and caused 85% reduction of viability for 10000 nM. The effectiveness of Dox-NPs is comparable to the effectiveness of doxorubicin, as doxorubicin reduced cell viability by 16% and 94% for concentrations 1000 nM and 10000 nM respectively.

## Discussion

### 4.1 Oxidized dextran can form NPs based on the coiling agent

Nanoparticles that are used in nanomedicine take advantage of the leakiness of blood vessels, which allows the flow of NPs with a diameter of up to 200 nm [22]. To meet this criterion, we performed NPs synthesis using combination of dextrans of different molecular weights with four degrees of oxidation and various types of coiling agents.

Dextran is a polymer that is composed of glucose units branched with alpha-1,6 and alpha-1,4 glycosidic bonds and can be oxidized to polyaldehydodextran by glucose ring opening without breaking the dextran backbone chain. To form NPs from dextran, we took advantage of the aldehyde groups that are present along dextran chains after oxidation and bound hydrophobic amines that serve as coiling agents, which allowed NPs formation by self-assembly in an aqueous environment.

Taking into account the additional criteria as NPs stability upon changing the substitution degree and lyophilization as well toxicity of a coiling agent we selected the combination of 70 kDa PAD with 5% oxidation degree and 55% degree of substitution with dodecylamine. The presence of remaining aldehyde groups allowed the attachment of the drug containing an amine group (by Schiff base formation).

### 4.2 Stable Dex-NP structure and composition

We have made an attempt to structurally characterize Dex-NPs with traditional scanning electron microscopy but no nanostructures were visible. Thus we decided to characterize Dex-NPs structure by the combination of the hydrodynamic diameter measurements and measurements performed with use of aerosol techniques. Comparisons of NPs diameters in the wet and dry state (Figs 3 and 6 respectively) revealed very high mass water content (> 95%) in Dex-NPs. This can explain the failure of the traditional SEM observation as the specimen preparation requires complete drying of the sample what in case of Dex-NPs can entirely destroy NPs structure. Eventually we were able to obtain structure visualization by exploitation of scanning electron microscope (SEM) SU8320 using ionic liquids. Thus we assume, that Dex-NPs do not form the regular structure like e.g. liposomal formulations, instead they self-assemble into a “skein” of dextran chains forming hydrogel structure of Dex-NP. Based on the results of aerosol analysis of Dex-NP we postulate that they are composed of approximately 10 modified dextran subunits and the change of substitution degree did not influence this number.

### 4.3 Storage stability

Another necessary feature for nanomedical use is the stability of NPs in solution and the possibility of long-term storage. Dex-NPs did not aggregate for at least 96 h in an aqueous solution (S2 Fig), which is sufficiently long for potential in patient application. Over longer periods of time, Dex-NPs can be stored in a lyophilized form. After rehydration, they regained their size distribution within 30 min. Moreover, lyophilization did not affect the pH-sensitive *in vitro* release rate of bound doxorubicin.

### 4.4 Drug release

One of the key factors that define an effective drug carrier is the release of entrapped drug at the site of action and the prevention of the burst effect [23]. In the case of Dox-NPs doxorubicin release rate is primarily limited by hydrolysis of the chemical bonds between the NPs and the drug. Assuming that NPs poses faintly organized water structure we can conclude that wide size distribution of Dox-NPs will be of little relevance on drug release. The results of the Dox-NPs release in two distinct release environments: in PBS and in the cell culture supported the phenomena observed in the previously reported research dextran-lipoic acid derivatives NPs [24] that cellular environment e.g. presence of enzymes, activity of endosomal trafficking, greatly accelerates the doxorubicin release. Thus, the doxorubicin release rates in the PBS can be used for monitoring of the pH influence on the drug release rate, whereas release time in PBS has limited predictive value for the estimation of time needed for a drug release intracellularly. No toxicity effect was observed for rDox-NPs which confirms reduction of connection between doxorubicin and NPs structure.

As the ultimate goal of the Dox-NPs is the doxorubicin release inside the human body the observation of doxorubicin release inside the living cell (Fig 8) justifies further nanosystem development for clinical use.

## Conclusions

We demonstrated that dextran chains modified with hydrophobic coiling agents can form NP backbone. Nanoparticles can be stored in a dry state and reassemble in water and reveal hydrogel structure. In water solution nanoparticles are in the equilibrium with modified dextran, like in the case of micelles and surfactant. We postulate that in order to achieve minimum structure energy modified dextran forms nanoparticles of approximately 10 dextran subunits per nanoparticle. Dex-NPs can serve as a carrier of drug (e.g., doxorubicin) with pH-dependent drug bonds that allow accelerated drug release with decreasing pH, also within cells. To be applicable in cancer nanotherapy, further experiments are needed to verify the targeting properties of Dex-NPs, but the present results indicate that dextran nanoparticles may be a promising drug nanocarrier.

## Supporting Information

S1 Fig Doxorubicin release rate in PBS. (A) Comparison of release of doxorubicin in free form and doxorubicin bound in NPs (lyophilized) in the PBS solution at two pHs (5.5 and 7.4) at 37°C. (B) Effect of NP lyophilization on the rate of doxorubicin release in PBS solution at pH 5.5 and 7.4 at 37°C. Mean n = 3 ± SD.
(TIF)

S2 Fig Aggregation of lyophilized and rehydrated Dox-NPs. Size distribution curve for the indicated time points. Aqueous Dox-NPs solution stored at 4°C, without stirring. Mean n = 3.
(TIF)

## References

[1] Pasteur L., Sur la fermentation visqueuse et la fermentation butyrique, 1. Bull Soc Chim. 1861; 30–31.

[2] Klemm D, Heinze T, editors. Polysaccharides II. Berlin New York: Springer; 2006.

[3] Wolf M, Koch TA, Bregman DB. Effects of iron deficiency anemia and its treatment on fibroblast growth factor 23 and phosphate homeostasis in women: FGF23 IN IRON DEFICIENCY. J Bone Miner Res. 2013;28: 1793–1803. 10.1002/jbmr.1923 23505057

[4] Alhareth K, Vauthier C, Bourasset F, Gueutin C, Ponchel G, Moussa F. Conformation of surface-decorating dextran chains affects the pharmacokinetics and biodistribution of doxorubicin-loaded nanoparticles. Eur J Pharm Biopharm. 2012;81: 453–457. 10.1016/j.ejpb.2012.03.009 22465096

[5] Anitha A, Deepagan VG, Divya Rani VV, Menon D, Nair SV, Jayakumar R. Preparation, characterization, in vitro drug release and biological studies of curcumin loaded dextran sulphate—chitosan nanoparticles. Carbohydr Polym. 2011;84: 1158–1164. 10.1016/j.carbpol.2011.01.005

[6] Li B, Wang Q, Wang X, Wang C, Jiang X. Preparation, drug release and cellular uptake of doxorubicin-loaded dextran-b-poly(ɛ-caprolactone) nanoparticles. Carbohydr Polym. 2013;93: 430–437. 10.1016/j.carbpol.2012.12.051 23499079

[7] Casadei MA, Cerreto F, Cesa S, Giannuzzo M, Feeney M, Marianecci C, et al Solid lipid nanoparticles incorporated in dextran hydrogels: A new drug delivery system for oral formulations. Int J Pharm. 2006;325: 140–146. 10.1016/j.ijpharm.2006.06.012 16846705

[8] Wu F, Zhou Z, Su J, Wei L, Yuan W, Jin T Development of dextran nanoparticles for stabilizing delicate proteins. Nanoscale Research Letters 2013;8:197 10.1186/1556-276X-8-197 23622054 PMC3655889

[9] Pasut G. Polymers for Protein Conjugation *Polymers* 2014; 6:160–178; 10.3390/polym6010160

[10] Yuan W, Geng Y, Wu F, Liu Y, Guo M, Zhao H, Jin T. Preparation of polysaccharide glassy microparticles with stabilization of proteins. Int J Pharma.2009;336:154–159 10.1016/j.ijpharm.2008.09.007 18835346

[11] Mehvar R. Dextrans for targeted and sustained delivery of therapeutic and imaging agents. J Control Release Off J Control Release Soc. 2000;69: 1–25. 10.1016/s0168-3659(00)00302-3 11018543

[12] Muangsiri W, Kirsch LE. The protein-binding and drug release properties of macromolecular conjugates containing daptomycin and dextran. Int J Pharm. 2006;315: 30–43. 10.1016/j.ijpharm.2006.02.016 16546333

[13] Fuentes M, Segura RL, Abian O, Betancor L, Hidalgo A, Mateo C, et al Determination of protein-protein interactions through aldehyde-dextran intermolecular cross-linking. Proteomics. 2004;4: 2602–2607. 10.1002/pmic.200300766 15352235

[14] Heindel ND, Zhao HR, Leiby J, VanDongen JM, Lacey CJ, Lima DA, et al Hydrazide pharmaceuticals as conjugates to polyaldehyde dextran: syntheses, characterization, and stability. Bioconjug Chem. 1990;1: 77–82. 1710144 10.1021/bc00001a010

[15] Szymanski WW, Bacher G, Allmaier G. Nano-aerosol approach for characterization of proteins and viruses In: Kish LB, Harvey EC, Spillman WB Jr, editors. 2001 pp. 38–44. 10.1117/12.454586

[16] Bacher G, Szymanski WW, Kaufman SL, Zöllner P, Blaas D, Allmaier G. Charge-reduced nano electrospray ionization combined with differential mobility analysis of peptides, proteins, glycoproteins, noncovalent protein complexes and viruses. J Mass Spectrom JMS. 2001;36: 1038–1052. 10.1002/jms.208 11599082

[17] Allmaier G, Laschober C, Szymanski WW. Nano ES GEMMA and PDMA, new tools for the analysis of nanobioparticles—Protein complexes, lipoparticles, and viruses. J Am Soc Mass Spectrom. 2008;19: 1062–1068. 10.1016/j.jasms.2008.05.017 18585927

[18] Ciach T, Diaz L, van den IJssel E, Marijnissen JCM. Application of Electro Hydro Dynamic Atomisation in the Production of Engineered Drug Particles In: Gradoń L, Marijnissen J, editors. Optimization of Aerosol Drug Delivery. Dordrecht: Springer Netherlands; 2003 pp. 189–204. Available: http://link.springer.com/10.1007/978-94-017-0267-6_11

[19] Maißer A, Attoui MB, Gañán-Calvo AM, Szymanski WW. Electro-hydrodynamic generation of monodisperse nanoparticles in the sub-10 nm size range from strongly electrolytic salt solutions: governing parameters of scaling laws. J Nanoparticle Res. 2013;15 10.1007/s11051-012-1318-2

[20] Lisman A, Butruk B, Wasiak I, Ciach T. Dextran/Albumin hydrogel sealant for Dacron(R) vascular prosthesis. J Biomater Appl. 2014;28: 1386–1396. 10.1177/0885328213509676 24221140

[21] Abdelwahed W, Degobert G, Stainmesse S, Fessi H. Freeze-drying of nanoparticles: formulation, process and storage considerations. Adv Drug Deliv Rev. 2006;58: 1688–1713. 10.1016/j.addr.2006.09.017 17118485

[22] Dobrovolskaia M, McNeil SE, editors. Handbook of immunological properties of engineered nanomaterials. [Hackensack] New Jersey: World Scientific; 2013.

[23] Yeo Y, Park K. Control of encapsulation efficiency and initial burst in polymeric microparticle systems. Arch Pharm Res. 2004;27: 1–12. 14969330 10.1007/BF02980037

[24] Li Y-L, Zhu L, Liu Z, Cheng R, Meng F, Cui J-H, et al Reversibly stabilized multifunctional dextran nanoparticles efficiently deliver doxorubicin into the nuclei of cancer cells. Angew Chem Int Ed Engl. 2009;48: 9914–9918. 10.1002/anie.200904260 19937876

