# Supplementary figures and images for: Dextran Nanoparticle Synthesis and Properties
Source: PLoS One. 2016 Jan 11;11(1):e0146237. doi: 10.1371/journal.pone.0146237 (PMC4713431; doi:10.1371/journal.pone.0146237)

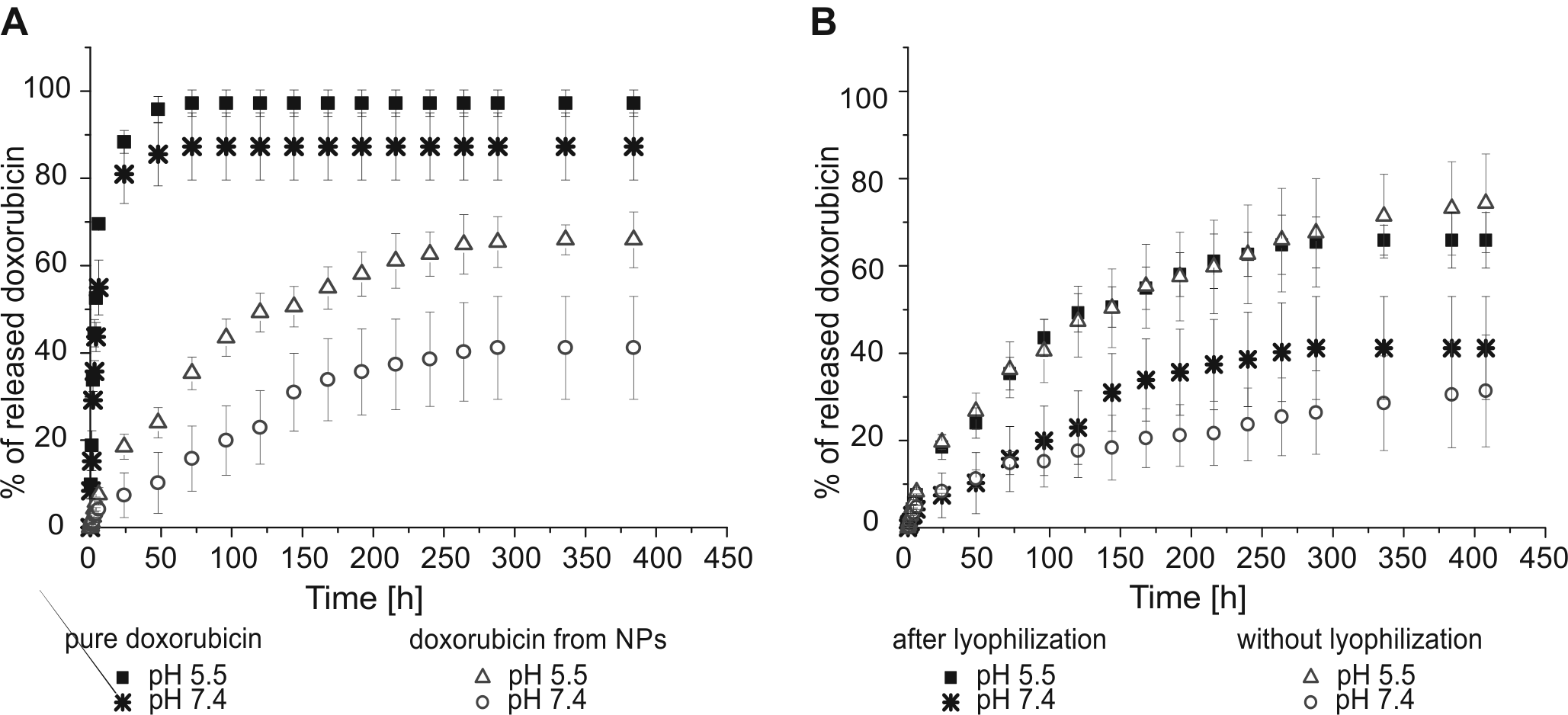

Supplement: S1 Fig — (A) Comparison of release of doxorubicin in free form and doxorubicin bound in NPs (lyophilized) in the PBS solution at two pHs (5.5 and 7.4) at 37°C. (B) Effect of NP lyophilization on the rate of doxorubicin release in PBS solution at pH 5.5 and 7.4 at 37°C. Mean n = 3 ± SD. (TIF) [file pone.0146237.s001.tif]

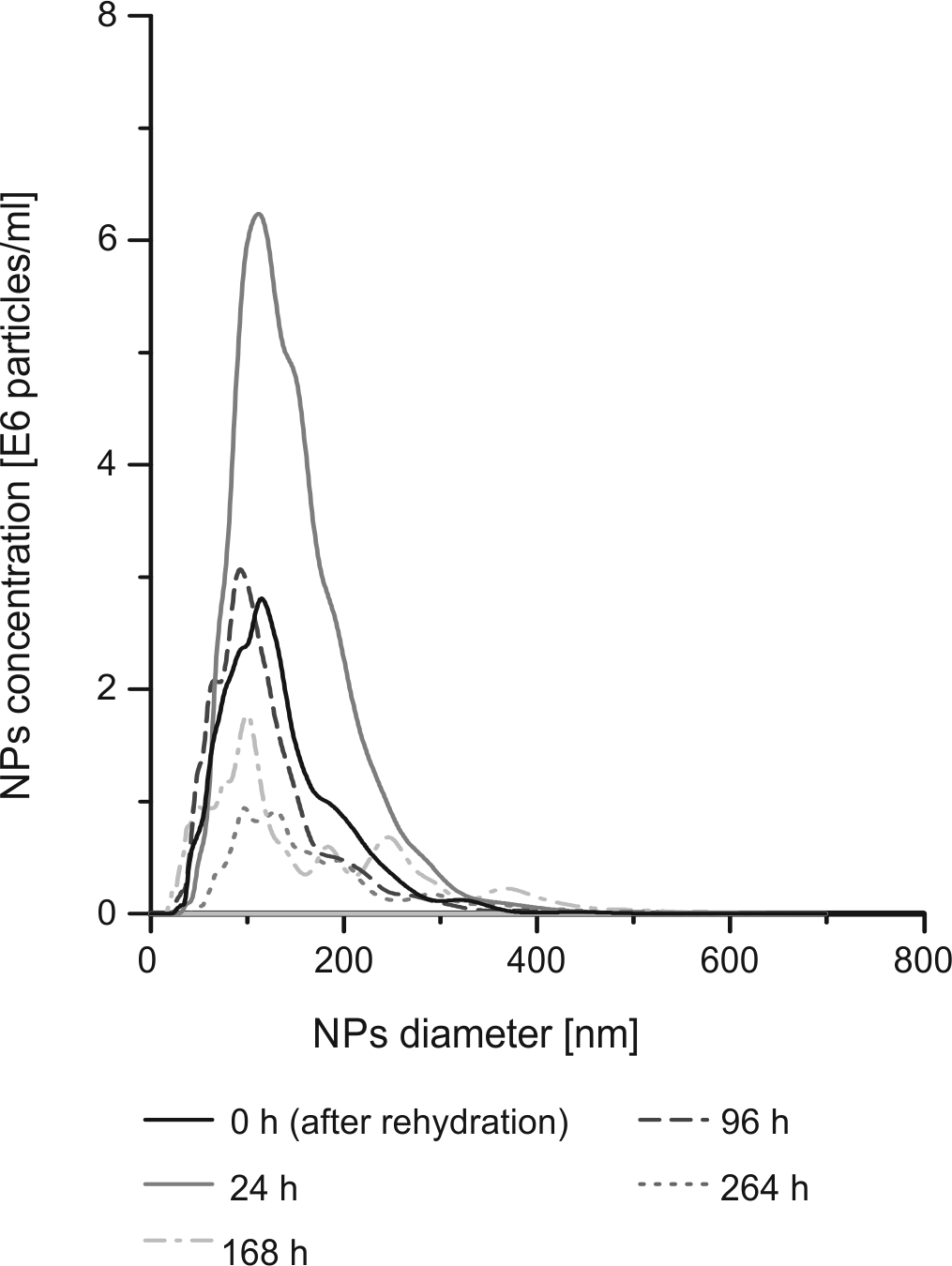

Supplement: S2 Fig — Size distribution curve for the indicated time points. Aqueous Dox-NPs solution stored at 4°C, without stirring. Mean n = 3. (TIF) [file pone.0146237.s002.tif]
